# Poverty, social exclusion, and mental health: the role of the family context in children aged 7–11 years INMA mother-and-child cohort study

**DOI:** 10.1007/s00787-021-01848-w

**Published:** 2021-07-26

**Authors:** Llúcia González, Marisa Estarlich, Mario Murcia, Florencia Barreto-Zarza, Loreto Santa-Marina, Sandra Simó, María Isabel Larrañaga, Estefanía Ruiz-Palomino, Jesús Ibarluzea, Marisa Rebagliato

**Affiliations:** 1 Joint Research Unit in Epidemiology, Environment and Health FISABIO-University of Valencia – Universitat Jaume I, Valencia, Spain; 2 Spanish Consortium for Research On Epidemiology and Public Health (CIBERESP), Instituto de Salud Carlos III, C/Monforte de Lemos 3-5, 28029 Madrid, Spain; 3 Department of Infirmary and Chiropody, University of Valencia, C/Jaume Roig s/n, 46010 Valencia, Spain; 4 Health Information Systems Analysis Service, Conselleria de Sanitat, Generalitat Valenciana, 46010 Valencia, Spain; 5 BIODONOSTIA Health Research Institute, Paseo Dr. Beguiristain, 20014 San Sebastian, Spain; 6 University of Basque Country, UPV/EHU, 48940 Leioa, Spain; 7 Public Health Division of Gipuzkoa, Basque Government, 4 Av. de Navarra, 20013 San Sebastian, Spain; 8 Department of Basic Psychology, University of Valencia, Av. Blasco Ibáñez, 21, 46010 Valencia, Spain; 9 Department of Basic and Clinical Psychology and Psychobiology, Universitat Jaume I, Av. Sos Baynat, 12071 Castelló de La Plana, Spain; 10 Predepartamental Unit of Medicine, Universitat Jaume I, Av. Sos Baynat, 12071 Castelló de La Plana, Spain

**Keywords:** Poverty, Family context, Internalizing problems, Externalizing problems

## Abstract

Mental health problems are common in childhood and tend to be more frequent in populations at risk of poverty or social exclusion (AROPE). The family environment can play a role in reducing the impact of economic hardship on these problems. The aim of this study was to assess the effect of multidimensional poverty on the mental health of children aged 7–11 years and the role of the family environment in two areas of Spain. Participants were 395 and 382 children aged 7 and 11 from Gipuzkoa and Valencia, respectively. Internalizing and externalizing problem scales of the child behaviour checklist (CBCL) were used. AROPE indicators were obtained by questionnaire, and three dimensions of the family context (Organization of the Physical Environment and Social Context, Parental Stress and Conflict, and Parental Profile Fostering Development) were measured through subscales 3, 4 and 5 of the Haezi-Etxadi family assessment scale (7–11) (HEFAS 7–11), respectively. Data were analysed using negative binomial regression and Structural Equation Modelling. AROPE prevalence was 7.1 and 34.5% in Gipuzkoa and Valencia, respectively. In both cohorts, there was a significant increase in internalizing and externalizing problems among participants with a higher AROPE score. However, AROPE did not affect internalizing problems in children from families living in a better physical environment and with social support (Subscale 3). The AROPE effect was jointly mediated by subscales 4 and 5 in 42 and 62% of internalizing and externalizing problems, respectively. Preventing economic inequities by economic compensation policies, improving the neighbourhood and immediate environment around the school, and promoting positive parenting programmes can improve mental health in childhood.

**Supplementary Information:**

The online version contains supplementary material available at 10.1007/s00787-021-01848-w.

## Introduction

Poverty and social exclusion are two concepts that describe people with scarce resources to have a dignified life, and those who have been separated from society [1]. Fighting poverty and social exclusion has always been a priority of the European Union (EU), which has typically measured these inequalities through the AROPE index (at risk of poverty or social exclusion). This index has been widely used in the Horizon 2020 programme [2], and in the 2030 Agenda [3], and it is composed of three sub-indicators: risk of poverty (based on household income), low work intensity (considering working hours) and severe material deprivation (such as not being able to afford certain goods or services). Meeting the conditions for at least one of the three sub-indicators implies being AROPE [2, 4]. In 2018, Spain had one of the highest rates of AROPE in the European Union (EU) (26.1%) [2, 5]. When assessing child poverty, the AROPE rate in Spain in 2018 differed depending on the type of family: children from two-parent families had a rate of 25.8%, while those from single-parent families presented a rate of 50% [6].

Children and adolescents are marked by critical periods of development, and not achieving a certain skill in a certain moment might have lifelong implications, even when remedial actions were implemented at later stages [7]. Socioeconomic inequalities may affect children’s development and mental health [8], which can be assessed through internalizing and externalizing problems. Internalizing, or emotional, problems are inward-directed symptoms that bring about suffering in the child [9]. They include anxiety, depression, somatic complaints and withdrawal [10], and their prevalence is around 8.7–22.6% in Spanish adolescents [11]. Several studies have shown adverse effects of economic hardship [12, 13], low socioeconomic status (SES) or parental education level [10, 14, 15] on internalizing problems [16, 17]. Externalizing, or behavioural, problems describe outward-directed symptoms that, in addition to producing suffering in the child, also cause discomfort in other people [9]. They comprise aggressive and oppositional behaviours, inattention/hyperactivity and emotion dysregulation [9], and their prevalence is around 2.4–14.6% in Spanish adolescents [11]. Poverty [13, 16, 17] and low parental education level [13, 15] were also associated with externalizing behaviours.

To understand how social inequalities affect mental health, Bronfenbrenner’s ecological systems theory (BEST), the family stress model (FSM) and the parental profile must be considered. According to the BEST [18], a child is the centre of concentric spheres of influence. Variations in the furthest structural determinants can affect children through family-specific factors such as parenting practices [14] or difficulties. Proximal social systems (family, school or community) can help promote the development of protective mechanisms that compensate the effect of unfavourable structural conditions [19–23]. Interventions on these proximal factors could be more feasible in the short term [24]. Stronger community, social and school networks have been positively related to better developmental outcomes [20, 21], even in children from lower socioeconomic positions [19, 20]. Assessing the role of these conditions is crucial to identify moderating factors. The importance of studying moderation (or effect modification) is clearly reflected in BEST, where community, social and school networks may temper or modulate the magnitude of the effect of socioeconomic strain on children’s mental health.

The FSM and the parental profile could describe a mediational pathway between economic strain and child’s mental health. The FSM posits that financial difficulties in the family generate stress that affects parenting practices, which may in turn influence child emotional and behavioural outcomes [25–27]. Several studies in this line have described how higher stress [17, 26, 28], maternal depression and harsh parenting [29] mediated internalizing and externalizing problems. Parental profile encompasses knowledge (for example, about developmental stages in children), attitudes (such as father’s involvement), beliefs (like environmentalist outlook on development), and feelings (parental self-efficacy), about parenting. Finally, the effect of parenting knowledge on children’s mental health has been explained as follows: parents in situation of poverty or social exclusion are more likely to have less knowledge about child development [30], and lower parenting self-efficacy (the confidence of doing well as parents) [31]. This could result in a poor parental profile and therefore lower quality parent–child interactions, thereby increasing the risk of children having socioemotional problems [32–34].

The INMA (INfancia y Medio Ambiente—Environment and Childhood) Study is a Spanish multicentre mother-and-child cohort [35]. Its main purpose is to describe how environmental conditions affect children’s growth and development. Previous analyses with our data found a social gradient in child cognitive development at the age of 1–2 years [36] and 5 years [37, 38] when SES indicators such as parental social class, educational level or employment status were used.

This work provides several novelties with respect to previous studies. Firstly, in comparison to socioeconomic indicators such as education, employment and social class, AROPE may have greater sensitivity to detect children at extreme risk, as it provides a deeper understanding of multidimensional poverty or exclusion. Secondly, epidemiological work focuses on socioeconomic inequalities and their impact on mental health, but rarely emphasizes the family and social environment as a key factor. One of the main strengths of this study is the fact that it provides a more comprehensive approach to the poverty–family–mental health pathway.

The first aim is to determine whether the family risk of poverty and social exclusion, as measured by the AROPE indicators, is related to internalizing and externalizing problems in children aged 7–11 from two regions in Spain (Gipuzkoa and Valencia), with distinct SES levels [2]. The second aim is to assess the dimensions of the family context that mediates or moderates the effect of poverty on children’s mental disorders. We hypothesize that: (a) children with a worse socioeconomic situation have a greater number of internalizing and externalizing problems, (b) parents’ stress caused by economic strain and the parental profile fostering child development act as a mediating pathway, and (c) the organization of the physical environment and social context acts as a moderating factor.

## Methods

### Study design and population

The INMA Study is a Spanish population-based mother-and-child multicentre cohort study set up in 2003 and is composed of seven cohorts (Ribera d’Ebre, Granada, Menorca, Valencia, Sabadell, Asturias and Gipuzkoa). This study uses data from the Valencia and Gipuzkoa cohorts. The recruitment process and subsequent procedures are described in more detail elsewhere [35]. Briefly, mothers were recruited during their first prenatal visit to their reference hospital before week 13 of gestation. The inclusion criteria were: at least 16 years of age, 10–13 weeks of gestation, singleton pregnancy, intention of undergoing follow-up and delivery at the corresponding centre of reference, and no impediment for communication. Eight hundred and fifty-five pregnant women were recruited in Valencia between November 2003 and June 2005, and six hundred and thirty-eight pregnant women were included in Gipuzkoa between May 2006 and February 2008. Follow-up visits were conducted at different ages of the children and the evolution of the sample due to withdrawals and losses during the follow-up is described in more detail elsewhere [4]. Data on the AROPE indicators of participating families were collected between 2014 and 2016 at the follow-up visits at 7–8 years and 11 years of age for Gipuzkoa and Valencia, respectively. Families included in this follow-up differed from those at recruitment, as in general terms, non-Spaniards or those who were the youngest parents, as well as having lower social class or education, were less likely to be included in the follow-up. Cohorts were approved by local institutional ethical review boards, and participants gave their consent to participate. This study conforms to the principles embodied in the Declaration of Helsinki.

### The AROPE indicators

The AROPE indicators were assessed by structured questionnaires self-completed by parents in their homes and revised by a trained interviewer at the follow-up visits at 7–11 years. AROPE has three sub-indicators that were calculated for each household [4]:

- 1) Low work intensity (LWI): having worked  < 20% of the hours available (for their members in working age).
- 2) At risk of poverty (RP): having  < 60% of Spanish median income per consumption unit.
- 3) Risk of material deprivation (MD): lacking ≥ 3 necessary items from a list of 9 [4].

AROPE [4, 38] were those households fulfilling at least one of the three sub-indicators mentioned above (LWI, RP or MD). In addition to the original dichotomous variables, we calculated a continuous AROPE score variable to obtain more precise results. We established continuous variables for each of the AROPE sub-indicators with a range of 0–1, where zero expressed the optimal condition (no risk) and one was the cut-off point used to define families at risk of each condition, as previously specified. Therefore:

- For low work intensity, families with a 100% work intensity obtained a score of 0 (no risk) and cases with work intensity lower than 20% were assigned a one. Intermediate values were linearly interpolated, i.e. a work intensity of 20%  < x < 100% was assigned a score of 1 − [(x−20)/(100−20)].
- For risk of poverty, we used the median income per consumption unit. Cases with an income lower than 60% of the Spanish median income per consumption unit were assigned a one. Cases with an income higher than the median were assigned a 0. Intermediate values were linearly interpolated, i.e. an income of 60% < x < 100% was assigned a score of 1 – [(x−60)/(100−60)].
- For material deprivation, the number of commodities lacking was considered and divided by three, resulting in a variable with a value of zero when there are no commodities lacking and one when there are three or more commodities lacking.
- Continuous AROPE was calculated by averaging these three continuous sub-indicators (min−max = 0−1).

Correlations between the AROPE original indicators and their corresponding AROPE continuous variables are represented in Supplementary material Fig S1.

### Internalizing and externalizing problems

Internalizing and externalizing problems were assessed by the child behaviour checklist [39]. This consists of a list of emotional and behavioural problems that must be answered by parents at the follow-up visits at 7–11 years, specifying whether the symptoms are not true (0), sometimes true (1) or always true (2). Its 113 items are distributed on nine syndrome scales: (1) Anxiety/depression, (2) withdrawal/depression, (3) somatic complaints, (4) social problems, (5) thought problems, (6) attention problems, (7) rule-breaking behaviour, (8) aggressive behaviour and (9) other problems. These scales can be summarized on two broadband scales: internalizing (composed of scales 1, 2 and 3) and externalizing (composed of scales 7 and 8) problems, with score ranges of (0–36) and (0–30), respectively. In this work, internalizing and externalizing raw scores were used adjusting for child’s age, sex and cohort in the statistical models.

### Family context

The HEFAS 7–11 was a questionnaire answered by parents at the follow-up visits at 7–11 years. It assessed the quality of the family context and parenting skills, and is an updated version of other traditional instruments such as the home observation for measurement of the environment (HOME) [40] and Pettit and Bates’ developmental history [41]. HEFAS 7–11 includes an update on family variables influencing child psychological development and it has been used and validated at the ages of 2 and 4 [42, 43], in 2014 it was updated and adapted to the age range 7–11 years [44]. An exploratory and confirmatory factor analysis was performed including participants from both cohorts, yielding five subscales [44] distributed in different factors including a total of 85 items to be answered using a six point Likert-type scale.

Subscales and factors are specified in the Supplemental material (Table S1) but, briefly, the subscales were the following: (1) Promotion of cognitive and linguistic development (PCLD), (2) promotion of social and emotional development (PSED), (3) organization of the physical environment and social context (OPESC); (4) parental stress and conflict (PSC) and (5) parental profile fostering child development (PPFCD). Ranges of weighted scores from all the subscales varied from 16.76 to 100, and higher scores on these subscales imply richer and more stimulating family contexts [44]. The scales have good internal consistency, with Cronbach’s alphas of 0.79, 0.80, 0.73, 0.75 and 0.75 (respectively). In this study, the weighted scores developed for each scale in a recent study [44] were used, rather than raw scores, to enable comparison across subscales. We tested our hypotheses employing the last three subscales mentioned above, hereinafter referred to as physical environment and social context, parental stress and parental profile, respectively, to check the BEST [19–23], FSM [25–27] and parental profile [30–34] models. We did not use the first two subscales on cognitive and emotional stimulation because we did not find any evidence relating them to both poverty and mental health problems.

### Covariates

Variables regarding family and parental characteristics as well as perinatal and child characteristics were collected by means of medical records and structured questionnaires at different follow-up visits (weeks 12 and 32 of pregnancy, birth and age 1, 5 and 11).

1. Family and parental characteristics: type of family (living with both parents/other combinations) and number of siblings at the age of the child’s evaluation. Parental age and country of origin (Spain/not Spain) were collected at pregnancy. Maternal and paternal tobacco use during pregnancy and at child’s evaluation (no/yes) and maternal alcohol consumption during pregnancy was also requested (no/yes). Parental mental health (no risk/at risk) and parental intelligence were measured at age 5 of the child. The latter was assessed using the similarities subtest of the Wechsler adult intelligence scale (WAIS-III) [45], as this subtest has been shown to be a good predictor of the overall IQ [45]. Parental mental health was assessed by “symptom checklist-90 revised” (SCL-90-R) [46]. Since the sample was not a clinical population, we identified the cases at risk of suffering a disorder as those who had a global severity index (GSI)  ≥ 1.5 standard deviations above the mean [46].

2. Perinatal and child characteristics: gender (male/female), age, parity (0/ ≥ 1), preterm birth (< 37 gestational weeks) (no/yes), and small for gestational age (SGA) (no/yes) were obtained from medical records. Duration of breastfeeding was collected at age 1.

### Statistical analysis

Proportions, medians and interquartile range were used in the descriptive analysis. For the bivariate analyses, Spearman correlations were used to assess the relationship between the CBCL scores and both the AROPE score and subscales 3, 4, and 5 of the HEFAS 7–11. Wald, Kruskal–Wallis and Mann–Whitney *U* tests were employed to assess the relationship of the covariates with the CBCL scores for categorical variables, and Spearman correlations for continuous variables.

The relationship between the AROPE score and CBCL was assessed by means of negative binomial regression models. This provided the incidence rate ratio (IRR), which can be interpreted as the % increase or decrease in internalizing or externalizing scores per one-unit change in the AROPE score, after adjusting for covariates. Cohort and child’s age and sex were included in all models regardless of their statistical significance. Several steps were performed sequentially: in the first step, multiple regression models were built considering covariates significantly related to the CBCL scores at *p* < 0.20 in the bivariate analyses and consecutively excluding those variables with a *p* value  > 0.10 in the adjusted model based on the likelihood ratio test. In the second step, the individual relationship between the AROPE score and the CBCL scales was assessed and possible confounders were tested (whether the effect estimates for the AROPE score changed by  ≥ 10% when they were included in the model).

In the third step, subscale 3 (Organization of the Physical Environment and Social Context, OPESC) from HEFAS 7–11 was evaluated as a potential confounder of the AROPE variable. Firstly, we performed a basal model adjusted for sex, age and cohort (model 0), secondly, we adjusted the previous model for other predictors and confounders (model 1), and thirdly we adjusted model 1 for subscale 3 (model 2). In the fourth step, the potential moderation of subscale 3 (OPESC) was assessed by adding an interaction term of the AROPE score and subscale 3 (OPESC) in the resulting model of the third step. To check separate trends of the AROPE score in the outcomes, we categorized subscale 3 (OPESC) by establishing cut-off points in tertiles and yielding groups of the lowest, middle and highest quality of context.

Finally, structural equation modelling (SEM) was performed to evaluate the mediating effect of subscale 4 (parental stress and conflict PSC) and subscale 5 (parental profile fostering child development PPFCD). To improve the fit of the models, a backwards procedure with likelihood ratio test (LRT) was performed to assess which other predictors and confounders related to the outcomes were also related to the potential mediators. In addition to simple mediation, simultaneous mediation of both variables was assessed. To perform the SEM analyses, the AROPE score, internalizing and externalizing problems, and subscales 4 and 5 from the HEFAS 7–11 were standardized [formula: *x*−(mean(*x*)/sd(*x*))]. Parameters were estimated using robust weighted least squares (WLSMV) and all SEM analyses presented good fit (comparative fit index, CFI  > 0.98 in all cases, and root mean square error approximation, RMSEA  < 0.048). Statistical analyses were carried out using SPSS, version 22.0 and R, version 3.5.1, SEM were fitted using the lavaan R package (Rosseel 2012), Fig. 1 was represented with the ggplot2 package and the rest of the figures were created with the open source diagram technology draw.io.

**Fig. 1:**
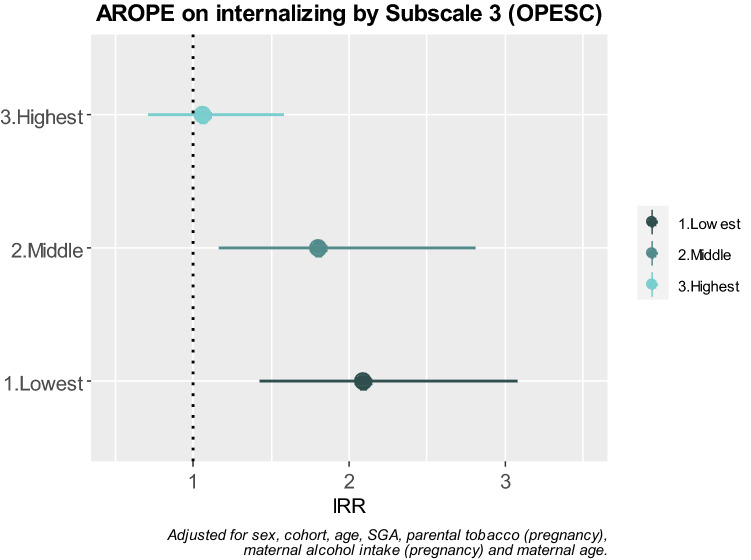
AROPE on internalizing problems stratified by quality of subscale 3 (OPESC)

## Results

### Descriptive analysis

Our analyses were completed with 394 and 382 participant families with mean (sd) years of age of the children of 7.76 (0.11) and 11 (0.32) in the Gipuzkoa and Valencia cohorts, respectively. Males and females were equally distributed across the two cohorts (50.3 and 51.8% of females in Gipuzkoa and Valencia, respectively). Information on sociodemographic characteristics can be found elsewhere [4] and in Table S3. Briefly, there were significant differences between cohorts in several factors. For instance, in comparison to Valencia, Gipuzkoa presented parents who were more frequently employed, native Spaniards and with higher social class and education. The median score (P25-P75) for Gipuzkoa and Valencia was 5 (2–9) and 6 (3–11) for internalizing problems (*p* < 0.001), and 5 (2–8) and 6 (2–10) for externalizing problems (*p* = 0.007), respectively. Table 1 shows the descriptive statistics of the HEFAS 7–11 scores and the AROPE score and their relation with internalizing and externalizing problems. The AROPE prevalence differed significantly by cohort (*p* < 0.001): rates were 34.5 and 7.1% for Valencia and Gipuzkoa, respectively. Higher AROPE scores were found in Valencia in comparison to Gipuzkoa, a median (p25–p75) of 0.31 (0.10–0.60) being reached in Valencia and 0.11 (0.03–0.23) in Gipuzkoa.

**Table 1:** AROPE score and HEFAS 7–11 subscales (Organization of the Physical Environment and Social Context, parental stress and conflict, and parental profile fostering child development) stratified by cohort

	GIPUZKOA							VALENCIA						
	Md^a^	P25	P75	Internalizing		Externalizing		Md^a^	P25	P75	Internalizing		Externalizing	
				Rho^b^	*p*^c^	Rho^b^	*p*^c^				Rho^b^	*p*^c^	Rho^b^	*p*^c^
AROPE score	0.11	0.03	0.23	0.20	< 0.001	0.20	< 0.001	0.31	0.10	0.60	0.19	< 0.001	0.18	0.001
Subscale 3 OPESC^d^	87.30	82.40	91.20	− 0.12	0.015	− 0.14	0.006	90.20	85.30	96.10	− 0.15	0.004	− 0.17	0.001
Subscale 4 PSC^e^	77.80	70.80	83.30	− 0.23	< 0.001	− 0.40	< 0.001	79.20	72.20	86.10	− 0.36	< 0.001	− 0.46	< 0.001
Subscale 5 PPFCD^f^	79.40	73.80	85.70	− 0.26	< 0.001	− 0.35	< 0.001	81.70	75.40	88.10	− 0.30	< 0.001	− 0.40	< 0.001

Correlation with internalizing and externalizing problems

^a^Md median

^b^Rho Spearman correlation coefficient

^c^p: *p* value from Spearman correlations

^d^OPESC: Organization of the Physical Environment and Social Context

^e^PSC: parental stress and conflict

^f^PPFCD: parental profile fostering child development

### Bivariate analyses

Table 1 shows the bivariate analyses. In both cohorts, the AROPE scores were directly related to internalizing and externalizing problems, with positive weak correlations: 0.19 and 0.18 for internalizing and externalizing problems in Valencia, and 0.20 for both outcomes in Gipuzkoa.

Physical environment and social context, parental stress and parental profile showed a significant inverse association for internalizing and externalizing scores meaning that higher quality of family context implied lower risk of internalizing and externalizing problems. The correlations for Gipuzkoa were weaker in comparison to those for Valencia. In the three subscales, correlations were stronger in externalizing scores. parental stress and parental profile presented weak to moderate associations, while physical environment and social context demonstrated weaker associations.

The relation between the covariates and the outcomes is depicted in Table S2*.* In both cohorts internalizing problems were related to parental tobacco use during pregnancy, maternal tobacco use at the 7–11 year follow-up and maternal mental health. Some factors were related only to Gipuzkoa or Valencia for internalizing problems. The cohort-related factors for Gipuzkoa were current paternal tobacco use, maternal alcohol intake during pregnancy and being SGA. In contrast, the cohort-related factors for these problems in Valencia were family type, parental age, maternal intelligence and parental mental health. Externalizing problems were related to maternal intelligence and mental health in both cohorts. Some factors were related only to Gipuzkoa or Valencia for externalizing problems. In Gipuzkoa, cohort-related factors were parental tobacco use during pregnancy, maternal alcohol intake during pregnancy, being SGA and parental mental health. In Valencia, cohort-related factors were family type, maternal tobacco use during pregnancy, paternal age and mental health.

### Multivariate analysis

The relation between the AROPE score and mental health problems are shown in Table 2. Model 0 displays estimators minimally adjusted for age, cohort and sex. Model 1 shows the results of model 0 adjusted for other predictors and confounders, and model 2 presents model 1 adjusted for physical environment and social context to test its confounder effect. Finally, an additional model was performed with an interaction term of the AROPE score with physical environment and social context to check the potential moderation effect.

**Table 2:** Incidence rate ratio of AROPE for internalizing and externalizing problems

	Internalizing				Externalizing			
	IRR^d^	95% CI		*p*	IRR^d^	95% CI		*p*
		Lower	Upper			Lower	Upper	
Model 0 minimally adjusted^a^	1.81	1.44	2.27	< 0.001	1.98	1.51	2.61	< 0.001
Model 1 adjusted for other predictors and confounders^b^	1.60	1.26	2.03	< 0.001	1.80	1.35	2.39	< 0.001
Model 2 adjusted for Subscale 3^c^	1.51	1.19	1.92	0.001	1.71	1.29	2.27	< 0.001

Predictors and confounders

Internalizing adjusted for: SGA, maternal and paternal tobacco in pregnancy, maternal alcohol in pregnancy and maternal age

Externalizing adjusted for: SGA, family type, paternal tobacco in pregnancy, maternal alcohol in pregnancy

^a^Model 0 adjusted for age, sex and cohort

^b^Model 1 model 0 + predictors and confounders

^c^Model 2 model 1 + subscale 3 (OPESC)

^d^*IRR* incidence risk ratio

The AROPE score showed a strong association with the CBCL scales after adjusting for age, cohort and sex (model 0), with significant risks in internalizing [IRR (95% CI) 1.81(1.44, 2.27)] and externalizing problems [IRR (95% CI) 1.98 (1.51, 2.61)]. A mild attenuation of the IRR of the AROPE in model 0 was observed when we adjusted it for other predictors and confounders (model 1), and when the potential confounding effect of physical environment and social context was checked (model 2). However, the associations remained statistically significant.

When the interaction of the AROPE score and physical environment and social context was tested, it appeared as statistically significant for internalizing (*p* interaction = 0.026) but not externalizing problems (*p* interaction = 0.656). To observe the functioning of this interaction, we stratified this subscale on three levels (lowest, middle and highest quality context) (Fig. 1). The AROPE score presented greater risks for internalizing problems on the two first levels of physical environment and social context [IRR (95% CI) 2.08(1.43, 3.08)] for the lowest quality, and 1.80 (1.16, 2.81) for the middle quality. However, on the highest quality level, no association was observed between the AROPE score and internalizing problems. No interaction by cohort was found in the multivariate analysis.

### Mediation analyses

#### Simple mediation

**Fig. 2:**
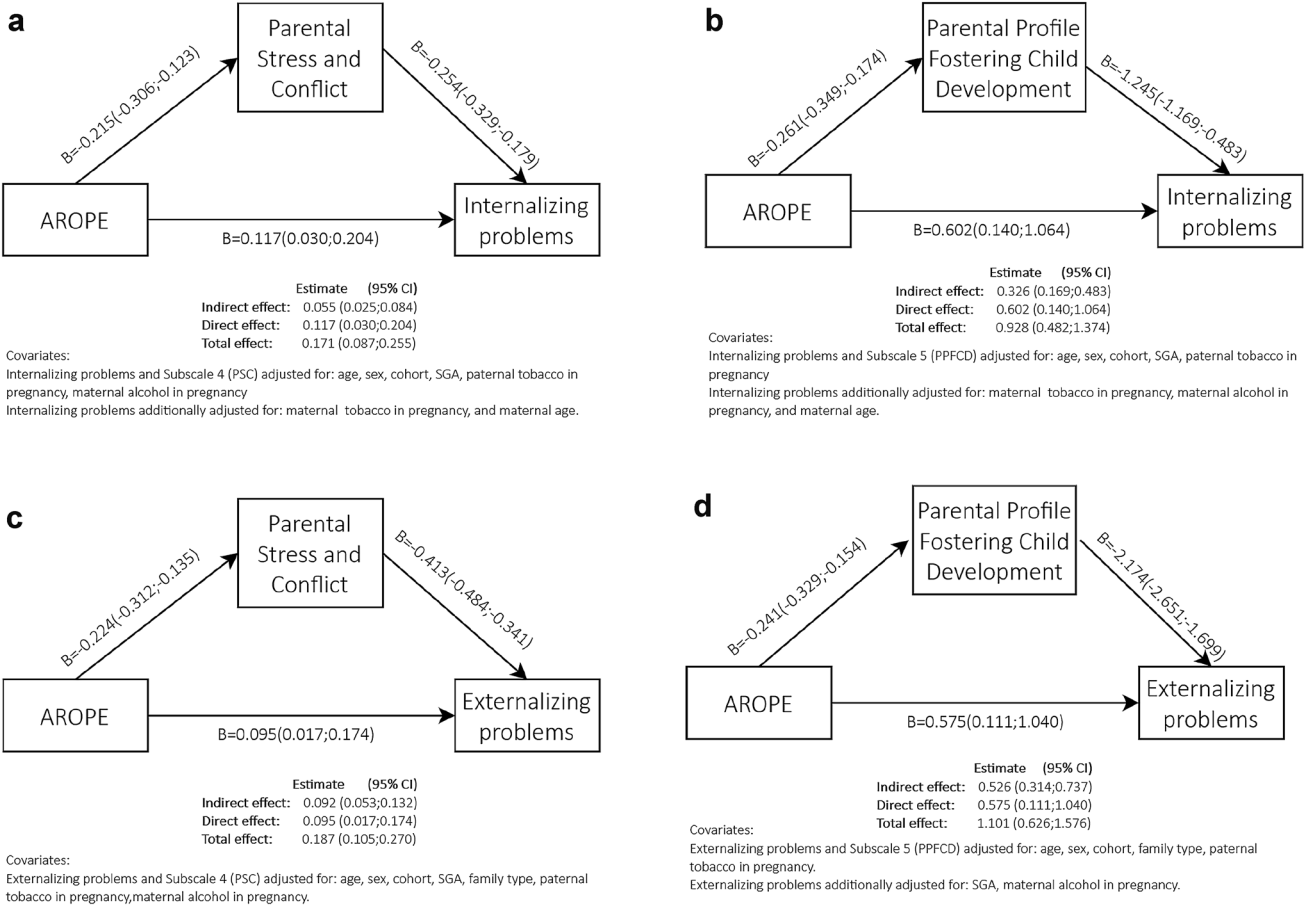
a) AROPE direct and indirect effect on internalizing problems mediated by subscale 4 (PSC). b) AROPE direct and indirect effect on internalizing problems mediated by subscale 5 (PPFCD). c) AROPE direct and indirect effect on externalizing problems mediated by subscale 4 (PSC). d) AROPE direct and indirect effect on externalizing problems mediated by subscale 5 (PPFCD)

To test the mediation paths as described in Fig. 2a–d, we performed SEM analyses. Figure 2a, b correspond to internalizing problems, and Fig. 2c, d correspond to externalizing problems. Figure 2a shows the mediator effect of parental stress. The total effect was 0.171 (CI 95% 0.087, 0.255) and the direct effect was 0.117 (CI 95% 0.030, 0.204), meaning that 32% of the total effect was mediated by parental stress. Similarly, Fig. 2b explores the mediation of parental profile, showing a total effect of 0.928 (CI 95% 0.482, 1.374) and a direct effect of 0.602 (CI 95% 0.140, 1.064), which implied that 35% of the total effect was mediated by parental profile.

When considering externalizing problems, the effects appeared to be similar to those in internalizing problems, with a slight decrease for the direct effect. Figure 2c assesses the mediator effect of parental stress. The total effect was 0.187 (CI 95% 0.105, 0.270) and the direct effect was 0.095 (CI 95% 0.017, 0.174), yielding a mediation of 49% of the total effect. In the same way, Fig. 2d shows the mediation of parental profile, with a total effect of 1.101 (CI 95% 0.626, 1.576) and a direct effect of 0.575 (CI 95% 0.111, 1.040), which implied that 48% of the total effect was mediated by parental profile.

### Simultaneous mediation

**Fig. 3:**
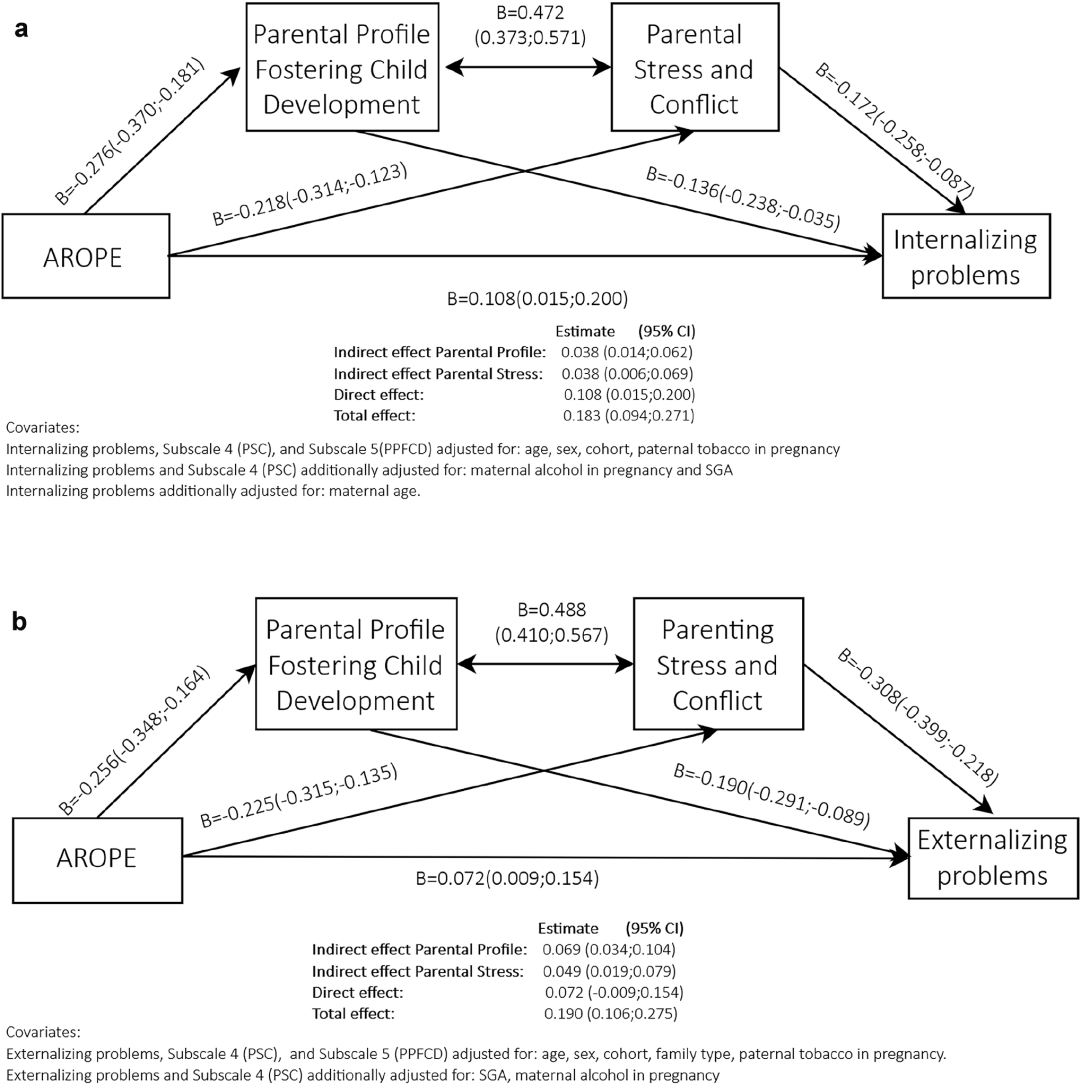
a) Simultaneous mediation AROPE direct and indirect effect on internalizing problems mediated by subscale 5 (PPFCD) and subscale 4 (PSC). b) Simultaneous mediation: AROPE direct and indirect effect on externalizing problems mediated by subscale 5 (PPFCD) and subscale 4 (PSC)

Simultaneous mediation was also evaluated (Fig. 3a, b) for both internalizing and externalizing problems. There was a direct effect of the AROPE score in the outcomes, and two indirect effects through parental profile and parental stress (Fig. 3a, b).

Figure 3a presented a total effect of 0.183 (CI 95% 0.094, 0.271) and a direct effect of 0.108 (CI 95% 0.015, 0.200), the indirect effects being evenly distributed between the two subscales. Parental profile and parental stress were strongly correlated to each other 0.472 (CI 95% 0.373, 0.571).

When comparing these results with those in Fig. 3b for externalizing problems, we observed a total effect in the same range as for Fig. 3a. However, the direct effect was slightly reduced in comparison to Fig. 3a. In consequence, indirect effects were greater. The indirect effects of the subscales were also more unevenly distributed, with greater weight for Parental profile [0.069 (CI 95% 0.034, 0.104)] in comparison to parental stress [0.049 (CI95% 0.019, 0.079)]. Correlation between subscales was slightly higher for externalizing problems: 0.488 (CI 95 0.410, 0.567).

These models yielded a mediation of 21% for internalizing problems through the two subscales, and a mediation of 36 and 26% for externalizing problems through parental profile and parental stress, respectively. All models showed a good fit (comparative fit index, CFI  > 0.98 in all cases, and root mean square error approximation, RMSEA  < 0.048). All the effects (total, direct and indirect) were significant in the six models presented.

## Discussion

We found that children from households at risk of poverty and exclusion and those with lower quality in the family context had higher scores for internalizing and externalizing problems. We also evaluated whether parental stress and parental profile were both mediators in the effect of poverty on children’s mental health, and if the physical environment and social context played a moderator role. Mediation analyses showed a direct and an indirect effect of risk of poverty and social exclusion on the outcomes, in both the simple and the simultaneous mediation, the latter demonstrating that both subscales can jointly mediate 42% of internalizing and 62% of externalizing problems. A moderation effect of the physical environment and social context was found for internalizing problems, with a positive relationship with the AROPE score in families with lower and middle quality on this subscale, while families with higher quality were not significantly affected by the AROPE. This fact suggests that a higher score on this subscale has a protective effect for poverty or social exclusion.

Several cohort studies have also explored behavioural outcomes in relation to family and community deprivation, such as the TRAILS Study [47], the ALSPAC Study [48] and the Millennium Cohort [16], which have widely depicted how youths with internalizing and externalizing problems are more frequently born to parents with a low social class or income, unemployment and primary education. The INMA study previously described the effect of gender and socioeconomic inequities on child cognitive development [37] and also analysed the factors associated with risk of poverty or social exclusion [4]. As far as we know, this is the first work to describe the relationship between poverty, family context and children’s mental health in a Spanish population [4].

Our work, as well as several other studies, has respected the layered structures to examine how socioeconomic hardship impacts children’s mental health. We found that both internalizing and externalizing problems were higher in more impoverished families and this relation was partially mediated by parental stress and parental profile. We did not find any studies with this mediation pattern, although some multi-level work has also described the effect of SES on mental health: one of them described how income inequality and family disruption were related to emotional problems [12]. The other two found that family poverty, parental stress and authoritative parenting were associated with poorer mental health in children [49, 50].

The parental stress and conflict subscale is composed of the factors of parental stress, frequency of and exposure to conflict, and conflict resolution. We found that greater AROPE was related to higher parental stress, and that this stress produced more risk of internalizing and externalizing problems. Three studies found trends compatible with our results [17, 25, 26]. One of these publications examined externalizing problems at two time points during childhood and found that economic hardship and pressure led to emotional distress, couple conflict, harsh parenting and externalizing problems [26]. We did not include some relational characteristics such as couple conflict or harsh parenting in our work, but our results point in the same direction as these findings. The second study tested to what extent two theories (family investment and family stress) explained the adverse relation between socioeconomic strain and externalizing problems. They found that the FSM was the pathway that best explained the relation between economic strain and adolescent delinquency, mediated by parents’ depression, caregiver conflict and parenting practices [25]. The third study is from the Millennium Cohort Study, and found that permanent income had a protective effect for children’s mental health. When reports were made by parents, this relation was mediated by maternal distress, but this did not occur when children’s mental health was reported by teachers [17]. This could represent a potential bias for the child’s psychopathology, and perhaps FSM is accountable only when both parental stress and the child’s behaviour are reported by parents. In addition to this problem, we must also keep in mind the potential reverse hypothesis: we argued that family stress was related to mental health problems, yet the child’s behaviour could be responsible for the parenting stress. A recent study found that in childhood, family stress was a predictor of externalizing, rather than the opposite, but in adolescence this relationship seems to be reciprocal [51].

In our study, parental profile was composed of factors such as parental self-efficacy, parental knowledge regarding development stages, assertiveness, theories on an environmentalist outlook on development, and father’s involvement. Our results showed that families with greater risk of poverty or exclusion had a poorer parental profile. This knowledge, feelings and attitudes about parenting presented an association with both internalizing and externalizing problems. We did not find any research describing the whole poverty–parental profile–mental health axis, but several publications did find that better parenting knowledge [30], self-efficacy [33] or parental involvement [52] reduced internalizing and externalizing problems.

Several studies have described the relation between parental stress and parental profile [31, 33, 53]. We chose a correlational approach between them to respect the two-way relation of these interdependent parenting characteristics [31]. We found a simultaneous mediation in both problems, but the magnitude and percentage of mediation was greater for externalizing problems. Parenting self-efficacy and parenting stress appear to be related [31, 54] and may be modulated through positive parenting programmes, as a reduction in stress and an increase in parenting self-efficacy have been observed in the short term [53], and an improvement in behaviour has been seen in the long term [33]. Some studies supported the evidence of our findings, as stress may reduce self-efficacy [54] and self-efficacy could predict parenting stress [55]. An Australian study considered children’s outcomes, and its aim was not to assess mental health-related factors, but to observe which factors were related to parental self-efficacy. This could be partially predicted (37%) when employing parenting stress, parental education and child’s mental health as predictors [31]. This study could be the most similar to ours, even when its hypothesis is reversed. They both have common factors, considering parental education (as a socioeconomic indicator), parenting stress, parental self-efficacy (as part of the parental profile) and the child’s mental health.

Organization of the Physical Environment and Social Context encompassed several related factors, such as quality of the physical environment, social support networks, promotion of child’s social relationships, and relations with the school. An Australian study described how community characteristics have a substantial impact on the child’s physical, mental and behavioural development, and more deprived areas have less appropriate neighbourhoods for children [21]. For instance, in the physical domain adverse behavioural outcomes are related to greater distance from green spaces and higher population density [21]. Another study examined the relations between socioeconomic characteristics and internalizing and externalizing problems, in a second factor: social support. This was inversely associated with both mental health problems in families and high socioeconomic status and low stress. This effect was not found for their low-status and high-stress counterparts, who reported greater behavioural problems in their children regardless of their social support [56]. We did not explore our data stratifying by socioeconomic position to observe the social support. Conversely, we did stratify physical environment and social context in tertiles to observe the AROPE risk in each stratum. Despite this methodological difference, both approaches rely on the fact that social support and socioeconomic position mutually influence each other. A third factor considered was the child’s social relationships, whereby it appears that having and keeping a best friend at childhood reduces mental health problems [57]. A final consideration in the Organization of the Physical Environment and Social Context is the interplay between school, friends and family. In particular, the relation of parents with the school in adverse environments is important. A recent study found that parental warmth and teacher support combined additively to reduce the effects of adversity in relation to internalizing problems [58].

Several limitations should be considered: first, there is the problem of representativeness. Due to sample attrition, conclusions might not be generalizable to other regions. Second, to check the family stress model, parental profile and the social context mechanisms more accurately, it would be necessary to measure variables that could have been overlooked, such as parental mental health or alcohol intake at evaluation time [17, 38]. Third, although we compared two cohorts that have proved to be substantially different, we did not find any interaction effect by cohort, and adding to the sample from other cohorts might help us to provide evidence that could be extrapolated to the general population in Spain. Fourth, income reports and family context scores could be biased, as many participants may have refused to answer when asked about their household’s income, and others could have masked family context answers for desirability. Fifth, the AROPE, HEFAS 7–11 and CBCL were reported at the same follow-up, a correlational rather than a causal relationship should be established [38, 52]. Sixth, parental characteristics such as stress and mental health could be biasing children’s symptoms, as they tend to over-report behaviour problems [17, 59]. Lastly, very few fathers answered the HEFAS 7–11 in comparison to mothers, so we could not stratify our analysis by respondent. However, no differences in subscales across respondents were observed, and results and significance did not change when we added the type of respondent, so simpler models were kept.

Our work also has several different strengths: first, we presented a new adaptation of the AROPE, to establish a continuous variable. This allowed us to increase the power of our analysis and provided richer information on the participating families and how much they are affected by poverty and exclusion. Second, we considered a full roster of covariates to improve the fit of our models and to control for potential confounders. Third, a strong measurement for family context, with good psychometric properties has been employed to describe the family characteristics. Fourth, to our knowledge, this is the first study to explore these characteristics as mediators between poverty and internalizing and externalizing problems, even though these factors have been interrelated in the literature. Lastly, the analysis was performed in two different cohorts with different social and cultural characteristics and with children ranging from 7 to 11 years of age: these facts endow our work with additional robustness.

By considering possible paths of intervention to ameliorate children’s symptoms, indirect and direct actions could be undertaken. Indirect interventions could consist in preventing economic inequities, eliminating the upstream causes of poverty itself, by economic compensation policies, such as providing a basic guaranteed income, implementing specific policies for single-parent families, reducing unemployment rates or increasing the minimum wage. These are proposals that are in line with the Spanish Government’s Strategy to fight poverty and exclusion [60], which were included as part of the agreement for forming the coalition government [61]. Proposals included in this agreement comprise the Minimum Vital Income, which was implemented in June 2020 [62, 63]. Future research will have to unveil the effectiveness of the Subsistence Income as a compensation mechanism.

Conversely, direct interventions are more related to families and the immediate environment around the school. These proximal and family factors could become the main asset for preventing the negative impact of socioeconomic disadvantage on children’s mental health problems, as positive parenting and community strategies may be implemented to foster the child’s wellbeing. This is in line with Recommendation 19 (2006) of the Council of Ministers of Europe to member states [64]. There is a need to invest in positive parenting programmes that can have a positive influence on children’s psychological development and indeed reduce the symptoms of internalizing and externalizing problems [24]. These programmes may mobilize parents to ask for more playgrounds, green areas or services in their neighbourhood. Positive parenting programmes could also promote relations with educational and health services, which might help to identify youths at risk of mental health problems. Education and health systems must provide parents with developmental knowledge to improve their parental self-efficacy. Finally, parenting programmes should offer them tools to promote hope and stress management to foster parent–child interactions [65]. In conclusion, preventing economic inequities by economic compensation policies such as the Subsistence Income, improving the neighbourhood and immediate environment around the school and social support, and promoting positive parenting programmes to strengthen parental self-efficacy could all improve mental health in childhood.

## Supplementary Information

Below is the link to the electronic supplementary material.

Supplementary file1 (DOCX 93 KB)
