# Supplementary material for: Poverty, social exclusion, and mental health: the role of the family context in children aged 7–11 years INMA mother-and-child cohort study
Source: Eur Child Adolesc Psychiatry. 2021 Jul 26;32(2):235–48. doi: 10.1007/s00787-021-01848-w (PMC9971086; doi:10.1007/s00787-021-01848-w)
Supplement: Supplementary file 1 — Supplementary file1 (DOCX 93 KB) [file 787_2021_1848_MOESM1_ESM.docx]

*Table S1:* Subscales and factors of HEFAS 7-11

| **Subscale 1: Promotion of Cognitive and Linguistic Development** | **(PCLD)** |
| --- | --- |
| 1.1.  Presence of Learning Materials | (PLM) |
| 1.2. Cognitive and Linguistic Scaffolding | (CLS) |
| 1.3. Encouraging Reading | (ER) |
| **Subscale 2: Promotion of Social and Emotional Development** | **(PSED)** |
| 2.1.  Emotional Expressiveness | (EE) |
| 2.2. Setting of Limits and Optimal Frustration | (SLOF) |
| 2.3. Fostering Autonomy and Self-esteem | (FAS) |
| 2.4. Precedents of Self-Regulated Learning | (PSRL) |
| 2.5. Quality of Sibling Relations | (QSR) |
| **Subscale 3: Organization of the Physical Environment and Social Context** | **(OPESC)** |
| 3.1.  Quality of the Physical Environment | (QPE) |
| 3.2. Social Support Networks | (SSN) |
| 3.3. Promotion of Child's Social Relationships | (PCSR) |
| 3.4. Relations with the School | (RS) |
| **Subscale 4: Parental Stress and Conflict** | **(PSC)** |
| 4.1.  Low Parental Stress | (LPS) |
| 4.2. Low Frequency of and Exposure to Conflict | (LFEC) |
| 3.3. Conflict Resolution | (CR) |
| **Subscale 5: Parental Profile Fostering Child Development** | **(PPFCD)** |
| 5.1.  Parental Self-Efficacy | (Au) |
| 5.2. Knowledge regarding Development | (KD) |
| 5.3. Assertiveness | (As) |
| 5.4. Environmentalist Outlook on Development | (EOD) |
| 5.5. Involvement of the Father or Secondary Reference Figure | (FI) |

*Table S2:* Internalizing and externalizing problems and related covariates stratified by cohort

|  | **BOTH COHORTS** | | | | | | | | **GIPUZKOA** | | | | | | **VALENCIA** | | | | | |
| --- | --- | --- | --- | --- | --- | --- | --- | --- | --- | --- | --- | --- | --- | --- | --- | --- | --- | --- | --- | --- |
|  | **Internalizing** | | | | **Externalizing** | | | | **Internalizing** | | | **Externalizing** | | | **Internalizing** | | | **Externalizing** | | |
|  | Md^a^ | IQR^b^ | p^c^ | | Md^a^ | IQR^b^ | p^c^ | | Md^a^ | IQR^b^ | p^d^ | Md^a^ | IQR^b^ | p^d^ | Md^a^ | IQR^b^ | p^d^ | Md^a^ | IQR^b^ | p^d^ |
| **Maternal country of origin** |  |  |  | |  |  |  | |  |  |  |  |  |  |  |  |  |  |  |  |
| Spain | 5.0 | 7.0 | 0.460 | | 5.0 | 7.0 | 0.772 | | 5.0 | 7.0 | 0.416 | 4.5 | 6.0 | 0.139 | 6.0 | 8.0 | 0.233 | 6.0 | 8.0 | 0.739 |
| Not Spain | 7.0 | 6.0 |  |  | 6.0 | 7.0 |  |  | 6.5 | 5.0 |  | 8.5 | 9.0 |  | 8.0 | 5.5 |  | 5.5 | 5.5 |  |
| **Paternal country of origin** |  |  |  | |  |  |  | |  |  |  |  |  |  |  |  |  |  |  |  |
| Spain | 5.0 | 6.0 | 0.497 | | 5.0 | 7.0 | 0.831 | | 5.0 | 7.0 | 0.175 | 5.0 | 6.0 | 0.609 | 6.0 | 8.0 | 0.426 | 6.0 | 8.0 | 0.984 |
| Not Spain | 7.0 | 8.0 |  |  | 6.0 | 7.0 |  |  | 9.0 | 8.0 |  | 5.5 | 5.0 |  | 7.0 | 8.0 |  | 6.0 | 7.0 |  |
| **Family type (2c)** |  |  |  | |  |  |  | |  |  |  |  |  |  |  |  |  |  |  |  |
| Both parents | 5.0 | 6.0 | 0.126 | | 5.0 | 7.0 | 0.077 | | 5.0 | 7.0 | 0.343 | 4.0 | 6.0 | 0.314 | 6.0 | 7.0 | 0.087 | 6.0 | 7.0 | 0.100 |
| Other combinations | 7.0 | 9.0 |  |  | 6.0 | 10.0 |  |  | 6.0 | 6.0 |  | 5.0 | 6.0 |  | 7.0 | 10.0 |  | 7.0 | 10.0 |  |
| **Main care provider** |  |  |  | |  |  |  | |  |  |  |  |  |  |  |  |  |  |  |  |
| Mother | 5.0 | 7.0 | 0.804 | | 5.0 | 7.0 | 0.969 | | 5.0 | 6.0 | 0.366 | 4.0 | 6.0 | 0.684 | 6.0 | 8.0 | 0.989 | 6.0 | 8.0 | 0.928 |
| Mother and others | 5.0 | 7.0 |  |  | 5.0 | 7.0 |  |  | 5.0 | 7.5 |  | 5.0 | 7.0 |  | 6.0 | 7.5 |  | 6.0 | 7.0 |  |
| Other combinations | 5.0 | 7.0 |  |  | 4.0 | 8.0 |  |  | 3.5 | 4.0 |  | 3.5 | 5.0 |  | 6.5 | 10.5 |  | 6.0 | 12.0 |  |
| **Number of siblings** |  |  |  | |  |  |  | |  |  |  |  |  |  |  |  |  |  |  |  |
| 0 | 6.0 | 8.0 | 0.512 | | 6.0 | 8.0 | 0.944 | | 6.5 | 8.0 | 0.192 | 7.5 | 8.0 | 0.165 | 6.0 | 9.0 | 0.719 | 6.0 | 8.0 | 0.878 |
| 1 | 5.0 | 6.0 |  |  | 5.0 | 7.0 |  |  | 5.0 | 7.0 |  | 4.0 | 6.0 |  | 6.5 | 7.0 |  | 6.0 | 8.0 |  |
| >1 | 5.0 | 6.0 |  |  | 5.0 | 8.0 |  |  | 4.0 | 6.0 |  | 4.0 | 7.5 |  | 5.0 | 8.0 |  | 6.0 | 7.0 |  |
| **Mother tobacco use in pregnancy** |  |  |  | |  |  |  | |  |  |  |  |  |  |  |  |  |  |  |  |
| No | 5.0 | 6.0 | 0.007 | | 5.0 | 7.0 | 0.050 | | 4.0 | 6.0 | 0.003 | 4.0 | 6.0 | 0.006 | 6.0 | 7.0 | 0.041 | 6.0 | 7.5 | 0.230 |
| Yes | 7.0 | 9.0 |  |  | 7.0 | 8.0 |  |  | 7.0 | 7.0 |  | 8.0 | 9.0 |  | 7.0 | 9.0 |  | 6.0 | 8.0 |  |
| **Father tobacco use in pregnancy** |  |  |  | |  |  |  | |  |  |  |  |  |  |  |  |  |  |  |  |
| No | 5.0 | 7.0 | 0.053 | | 5.0 | 7.0 | 0.030 | | 4.0 | 6.0 | 0.069 | 4.0 | 7.0 | 0.017 | 5.0 | 7.0 | 0.012 | 5.0 | 7.0 | 0.137 |
| Yes | 7.0 | 8.0 |  |  | 6.0 | 7.0 |  |  | 5.0 | 7.0 |  | 6.0 | 7.5 |  | 7.0 | 9.0 |  | 6.0 | 7.0 |  |
| **Mother present tobacco use** |  |  |  | |  |  |  | |  |  |  |  |  |  |  |  |  |  |  |  |
| No | 5.0 | 7.0 | 0.053 | | 5.0 | 7.0 | 0.065 | | 5.0 | 6.0 | 0.074 | 4.0 | 6.0 | 0.127 | 6.0 | 7.0 | 0.018 | 5.0 | 7.0 | 0.008 |
| Yes | 7.0 | 8.0 |  |  | 6.0 | 8.0 |  |  | 6.0 | 7.0 |  | 5.0 | 7.0 |  | 7.0 | 8.0 |  | 7.0 | 7.0 |  |
| **Father present tobacco use** |  |  |  | |  |  |  | |  |  |  |  |  |  |  |  |  |  |  |  |
| No | 5.0 | 6.0 | 0.366 | | 5.0 | 7.0 | 0.278 | | 4.0 | 6.0 | 0.071 | 5.0 | 6.0 | 0.431 | 6.0 | 8.0 | 0.458 | 6.0 | 8.0 | 0.373 |
| Yes | 7.0 | 7.0 |  |  | 6.0 | 7.0 |  |  | 5.0 | 7.0 |  | 4.0 | 6.5 |  | 7.0 | 7.0 |  | 7.0 | 7.0 |  |
|  | **Internalizing** | | | | **Externalizing** | | | | **Internalizing** | | | **Externalizing** | | | **Internalizing** | | | **Externalizing** | | |
|  | Md^a^ | IQR^b^ | p^c^ | | Md^a^ | IQR^b^ | p^c^ | | Md^a^ | IQR^b^ | p^d^ | Md^a^ | IQR^b^ | p^d^ | Md^a^ | IQR^b^ | p^d^ | Md^a^ | IQR^b^ | p^d^ |
| **Maternal alcohol intake in pregnancy** |  |  |  | |  |  |  | |  |  |  |  |  |  |  |  |  |  |  |  |
| No | 5.0 | 7.0 | 0.129 | | 5.0 | 7.0 | 0.055 | | 4.0 | 5.0 | 0.002 | 4.0 | 5.0 | 0.017 | 6.0 | 8.0 | 0.828 | 6.0 | 8.0 | 0.814 |
| Yes | 6.0 | 7.0 |  |  | 6.0 | 7.0 |  |  | 5.0 | 7.0 |  | 5.0 | 7.0 |  | 6.0 | 8.0 |  | 6.0 | 7.0 |  |
| **Breastfeeding (weeks)** |  |  |  | |  |  |  | |  |  |  |  |  |  |  |  |  |  |  |  |
| 0 | 5.0 | 7.0 | 0.577 | | 5.0 | 7.0 | 0.489 | | 5.0 | 4.0 | 0.837 | 3.5 | 7.0 | 0.989 | 6.0 | 7.0 | 0.360 | 6.0 | 8.0 | 0.284 |
| >0-16 | 6.0 | 7.0 |  |  | 6.0 | 7.0 |  |  | 5.0 | 6.0 |  | 5.0 | 7.0 |  | 7.0 | 7.0 |  | 6.0 | 8.5 |  |
| >16-24 | 5.0 | 9.0 |  |  | 5.5 | 8.5 |  |  | 4.0 | 8.0 |  | 4.0 | 6.0 |  | 6.0 | 9.0 |  | 7.0 | 10.0 |  |
| >24 | 5.0 | 7.0 |  |  | 5.0 | 6.0 |  |  | 5.0 | 6.0 |  | 5.0 | 6.0 |  | 6.0 | 7.0 |  | 5.0 | 7.0 |  |
| **Preterm (<37 weeks)** |  |  | |  | |  |  |  |  |  |  |  |  |  |  |  |  |  |  |  |
| No | 5.0 | 7.0 | | 0.923 | | 5.0 | 7.0 | 0.715 | 5.0 | 7.0 | 0.461 | 5.0 | 6.0 | 0.939 | 6.0 | 8.0 | 0.911 | 6.0 | 8.0 | 0.915 |
| Yes | 7.0 | 7.0 | |  |  | 4.5 | 6.0 |  | 7.0 | 8.0 |  | 5.0 | 5.0 |  | 7.0 | 7.0 |  | 4.0 | 6.0 |  |
| **SGA** |  |  | |  | |  |  |  |  |  |  |  |  |  |  |  |  |  |  |  |
| No | 5.0 | 6.0 | | 0.074 | | 5.0 | 7.0 | 0.051 | 4.5 | 6.0 | 0.013 | 4.0 | 6.0 | 0.077 | 6.0 | 7.0 | 0.509 | 6.0 | 8.0 | 0.503 |
| Yes | 7.5 | 8.0 | |  |  | 7.0 | 11.5 |  | 8.5 | 10.0 |  | 7.0 | 12.0 |  | 7.0 | 8.0 |  | 6.0 | 10.0 |  |
| **Child's sex** |  |  | |  | |  |  |  |  |  |  |  |  |  |  |  |  |  |  |  |
| Female | 5.0 | 6.0 | | 0.282 | | 5.0 | 6.0 | 0.017 | 5.0 | 6.0 | 0.604 | 5.0 | 6.0 | 0.254 | 6.0 | 7.0 | 0.177 | 5.0 | 7.0 | 0.069 |
| Male | 6.0 | 7.0 | |  |  | 5.0 | 8.0 |  | 5.0 | 7.0 |  | 4.5 | 7.0 |  | 7.0 | 8.0 |  | 7.0 | 8.0 |  |
|  | **Internalizing** | | | | | **Externalizing** | | | **Internalizing** | | | **Externalizing** | | | **Internalizing** | | | **Externalizing** | | |
|  | **Rho^e^** | | | **p^f^** | | **Rho^e^** | | **p^f^** | **Rho^e^** | | **p^f^** | **Rho^e^** | | **p^f^** | **Rho^e^** | | **p^f^** | **Rho^b^** | | **p^e^** |
| Child’s age | 0.09 | | | 0.010 | | 0.06 | | 0.106 | 0.05 | | 0.302 | 0.01 | | 0.808 | -0.13 | | 0.014 | -0.12 | | 0.019 |
| Maternal age | 0.05 | | | 0.173 | | 0.04 | | 0.214 | -0.07 | | 0.165 | -0.06 | | 0.221 | -0.13 | | 0.013 | -0.07 | | 0.192 |
| Paternal age | 0.03 | | | 0.370 | | 0.04 | | 0.227 | -0.02 | | 0.739 | 0.03 | | 0.561 | -0.21 | | <0.001 | -0.16 | | 0.001 |
| Maternal intelligence | -0.07 | | | 0.056 | | -0.14 | | <0.001 | -0.04 | | 0.494 | -0.11 | | 0.059 | -0.12 | | 0.028 | -0.18 | | 0.001 |
| Paternal intelligence | -0.14 | | | 0.156 | | -0.14 | | 0.151 | NA | | NA | NA | | NA | -0.14 | | 0.156 | -0.14 | | 0.151 |
| Maternal mental health | 0.33 | | | <0.001 | | 0.28 | | <0.001 | 0.27 | | <0.001 | 0.33 | | <0.001 | 0.37 | | <0.001 | 0.26 | | <0.001 |
| Paternal mental health | 0.16 | | | <0.001 | | 0.12 | | 0.008 | 0.11 | | 0.098 | 0.12 | | 0.074 | 0.21 | | <0.001 | 0.13 | | 0.024 |

^a^: Median

^b^: Inter-Quartile Range

^c^: p-value from Kruskal-Wallis test

^d^: p-value from Wald test adjusted by cohort

^e^: Spearman correlation coefficient

^f^: p-value from Spearman correlations

*Figure S1:* Original AROPE and AROPE score correlation

Table S3: Sample characteristics stratified by cohort

|  |  | Both cohorts | | Gipuzkoa | | Valencia | | p value^a^ |
| --- | --- | --- | --- | --- | --- | --- | --- | --- |
|  | | N | % | N | % | N | % |  |
| Maternal occupation | Employed | 585 | 76.67 | 324 | 83.29 | 261 | 69.79 | <0.001 |
|  | Homemaker | 73 | 9.57 | 29 | 7.46 | 44 | 11.76 |  |
|  | Short-term unemployment | 38 | 4.98 | 11 | 2.83 | 27 | 7.22 |  |
|  | Long-term unemployment | 58 | 7.60 | 18 | 4.63 | 40 | 10.70 |  |
|  | Others | 9 | 1.18 | 7 | 1.80 | 2 | 0.53 |  |
| Paternal occupation | Employed | 634 | 92.83 | 360 | 95.74 | 274 | 89.25 | 0.013 |
|  | Homemaker | 1 | 0.15 | 1 | 0.27 | 0 | 0.00 |  |
|  | Short-term unemployment | 21 | 3.07 | 7 | 1.86 | 14 | 4.56 |  |
|  | Long-term unemployment | 20 | 2.93 | 6 | 1.60 | 14 | 4.56 |  |
|  | Others | 7 | 1.02 | 2 | 0.53 | 5 | 1.63 |  |
| Maternal country of origin | Native | 745 | 96.01 | 384 | 97.46 | 361 | 94.50 | 0.035 |
|  | Non-native | 31 | 3.99 | 10 | 2.54 | 21 | 5.50 |  |
| Paternal country of origin | Native | 728 | 93.94 | 388 | 98.48 | 340 | 89.24 | <0.001 |
|  | Non-native | 47 | 6.06 | 6 | 1.52 | 41 | 10.76 |  |
| Maternal education | Up to primary | 134 | 17.31 | 40 | 10.20 | 94 | 24.61 | <0.001 |
|  | Secondary | 305 | 39.41 | 142 | 36.22 | 163 | 42.67 |  |
|  | University | 335 | 43.28 | 210 | 53.57 | 125 | 32.72 |  |
| Paternal education | Up to primary | 238 | 30.91 | 82 | 21.03 | 156 | 41.05 | <0.001 |
|  | Secondary | 336 | 43.64 | 190 | 48.72 | 146 | 38.42 |  |
|  | University | 196 | 25.45 | 118 | 30.26 | 78 | 20.53 |  |
| Maternal social class | Higher | 213 | 27.45 | 132 | 33.50 | 81 | 21.20 | <0.001 |
|  | Middle | 222 | 28.61 | 114 | 28.93 | 108 | 28.27 |  |
|  | Lower | 341 | 43.94 | 148 | 37.56 | 193 | 50.52 |  |
| Paternal social class | Higher | 184 | 23.77 | 111 | 28.24 | 73 | 19.16 | 0.002 |
|  | Middle | 134 | 17.31 | 54 | 13.74 | 80 | 21.00 |  |
|  | Lower | 456 | 58.91 | 228 | 58.02 | 228 | 59.84 |  |
| Type of family | Nuclear | 666 | 86.27 | 364 | 92.86 | 302 | 79.47 | <0.001 |
|  | Mother with another partner | 58 | 7.51 | 15 | 3.83 | 43 | 11.32 |  |
|  | Single-mother | 43 | 5.57 | 13 | 3.32 | 30 | 7.89 |  |
|  | Others | 5 | 0.65 | 0 | 0.00 | 5 | 1.32 |  |
| Number of siblings | 0 | 144 | 18.73 | 36 | 9.30 | 108 | 28.27 | <0.001 |
|  | 1 | 505 | 65.67 | 279 | 72.09 | 226 | 59.16 |  |
|  | >1 | 120 | 15.60 | 72 | 18.60 | 48 | 12.57 |  |
| Main care provider | 1 | 407 | 56.45 | 172 | 49.14 | 235 | 63.34 | <0.001 |
|  | 2 | 248 | 34.40 | 144 | 41.14 | 104 | 28.03 |  |
|  | 3 | 66 | 9.15 | 34 | 9.71 | 32 | 8.63 |  |
| Mother tobacco use in pregnancy | No | 642 | 83.92 | 342 | 89.30 | 300 | 78.53 | <0.001 |
|  | Yes | 123 | 16.08 | 41 | 10.70 | 82 | 21.47 |  |
| Father tobacco use in pregnancy | No | 490 | 64.05 | 283 | 73.89 | 207 | 54.19 | <0.001 |
|  | Yes | 275 | 35.95 | 100 | 26.11 | 175 | 45.81 |  |
| Mother present tobacco use | No | 585 | 75.58 | 322 | 81.93 | 263 | 69.03 | <0.001 |
|  | Yes | 189 | 24.42 | 71 | 18.07 | 118 | 30.97 |  |
|  | | Both cohorts | | Gipuzkoa | | Valencia | | p value^a^ |
|  |  | N | % | N | % | N | % |  |
| Father present tobacco use | No | 577 | 74.84 | 298 | 76.41 | 279 | 73.23 | 0.309 |
|  | Yes | 194 | 25.16 | 92 | 23.59 | 102 | 26.77 |  |
| Maternal alcohol intake in pregnancy | No | 371 | 49.01 | 162 | 42.19 | 209 | 56.03 | <0.001 |
|  | Yes | 386 | 50.99 | 222 | 57.81 | 164 | 43.97 |  |
| Breastfeeding (weeks) | 0 | 83 | 10.99 | 32 | 8.58 | 51 | 13.35 | 0.102 |
|  | >0-16 | 161 | 21.32 | 77 | 20.64 | 84 | 21.99 |  |
|  | >16-24 | 128 | 16.95 | 61 | 16.35 | 67 | 17.54 |  |
|  | >24 | 383 | 50.73 | 203 | 54.42 | 180 | 47.12 |  |
| Preterm (<37 weeks) | No | 741 | 95.86 | 377 | 96.17 | 364 | 95.54 | 0.657 |
|  | Yes | 32 | 4.14 | 15 | 3.83 | 17 | 4.46 |  |
| SGA | No | 695 | 90.61 | 356 | 92.23 | 339 | 88.98 | 0.123 |
|  | Yes | 72 | 9.39 | 30 | 7.77 | 42 | 11.02 |  |
| Child's sex | Female | 396 | 51.03 | 198 | 50.25 | 198 | 51.83 | 0.660 |
|  | Male | 380 | 48.97 | 196 | 49.75 | 184 | 48.17 |  |
|  |  | Md^b^ | IQR^c^ | Md^b^ | IQR^c^ | Md^b^ | IQR^c^ | p value^d^ |
| Child’s age | | 8.05 | 3.24 | 7.74 | 0.07 | 10.98 | 0.24 | <0.001 |
| Maternal age | | 37 | 11.75 | 31 | 4.00 | 43 | 5.66 | <0.001 |
| Paternal age | | 39 | 11.36 | 33 | 6.00 | 44 | 6.25 | <0.001 |
| Maternal intelligence | | 9.76 | 3.67 | 9.76 | 3.67 | 10.50 | 3.67 | 0.573 |
| Paternal intelligence | | 9.13 | 3.57 | NA | NA | 9.13 | 3.57 | NA |
| Maternal mental health | | 48.69 | 12.03 | 48.46 | 11.39 | 48.93 | 11.90 | 0.326 |
| Paternal mental health | | 48.19 | 14.42 | 49.61 | 16.40 | 47.34 | 12.16 | 0.082 |

^a^: p value from chi-square test

^b^: Md: Median

^c^: IQR: Inter-Quartile Range

^d^: p-value from Kruskal-Wallis test

Table S4: Differences between included and not included

|  | | Gipuzkoa | | | | | Valencia | | | | |
| --- | --- | --- | --- | --- | --- | --- | --- | --- | --- | --- | --- |
|  |  | Whole sample at recruitment | | Visit at 7–11 years | | | Whole sample at recruitment | | Visit at 7–11 years | | |
|  |  | n | % | Not included (%) | Included (%) | p^a^ | n | % | Not included (%) | Included (%) | p^a^ |
| Maternal country of origin | Spain | 610 | 95.61 | 92.74 | 97.44 | 0.005 | 728 | 88.03 | 82.55 | 94.47 | <0.001 |
|  | Not Spain | 28 | 4.39 | 7.26 | 2.56 |  | 99 | 11.97 | 17.45 | 5.53 |  |
| Paternal country of origin | Spain | 612 | 95.90 | 91.90 | 98.5 | <0.001 | 705 | 85.20 | 82.10 | 88.9 | <0.001 |
|  | Not Spain | 26 | 4.10 | 8.10 | 1.50 |  | 122 | 14.8 | 17.90 | 11.10 |  |
| Maternal age at birth | <25 | 16 | 2.51 | 5.24 | 0.77 | 0.400 | 108 | 12.63 | 17.26 | 6.84 | 0.008 |
|  | 25–29 | 193 | 30.25 | 29.84 | 30.51 |  | 298 | 34.85 | 37.05 | 32.11 |  |
|  | 30–34 | 310 | 48.59 | 43.95 | 51.54 |  | 323 | 37.78 | 33.05 | 43.68 |  |
|  | 35+ | 119 | 18.65 | 20.97 | 17.18 |  | 126 | 14.74 | 12.63 | 17.37 |  |
| Paternal age at birth | <26 | 11 | 1.73 | 2.42 | 1.29 | 0.001 | 78 | 9.47 | 12.39 | 6.05 | <0.001 |
|  | 26–30 | 148 | 23.23 | 25.81 | 21.59 |  | 259 | 31.43 | 32.43 | 30.26 |  |
|  | 31–35 | 278 | 43.64 | 41.13 | 45.24 |  | 303 | 36.77 | 33.78 | 40.26 |  |
|  | 36+ | 200 | 31.40 | 30.65 | 31.88 |  | 184 | 22.33 | 21.40 | 23.42 |  |
| Maternal social class | Highest | 193 | 30.25 | 25.40 | 33.33 | 0.012 | 128 | 15.48 | 10.51 | 21.32 | <0.001 |
|  | Middle | 173 | 27.12 | 22.98 | 29.74 |  | 196 | 23.70 | 20.13 | 27.89 |  |
|  | Lowest | 272 | 42.63 | 51.61 | 36.92 |  | 503 | 60.82 | 69.35 | 50.79 |  |
| Paternal social class | Highest | 158 | 25.16 | 19.17 | 28.87 | 0.006 | 110 | 13.40 | 8.39 | 19.21 | <0.001 |
|  | Middle | 82 | 13.06 | 12.08 | 13.66 |  | 158 | 19.24 | 17.91 | 20.79 |  |
|  | Lowest | 388 | 61.78 | 68.75 | 57.47 |  | 550 | 66.99 | 73.24 | 59.74 |  |
| Maternal education level | Up to primary | 86 | 13.52 | 18.62 | 10.28 | <0.001 | 288 | 34.82 | 43.40 | 24.74 | <0.001 |
|  | Secondary | 232 | 36.48 | 36.84 | 36.25 |  | 351 | 42.44 | 42.28 | 42.63 |  |
|  | University | 318 | 50.00 | 44.53 | 53.47 |  | 188 | 22.73 | 14.32 | 32.63 |  |
| Paternal education level | Up to primary | 162 | 25.63 | 32.93 | 20.98 | 0.740 | 387 | 47.08 | 52.03 | 41.27 | 0.546 |
|  | Secondary | 304 | 48.10 | 47.56 | 48.45 |  | 315 | 38.32 | 38.51 | 38.10 |  |
|  | University | 166 | 26.27 | 19.51 | 30.57 |  | 120 | 14.60 | 9.46 | 20.63 |  |

^a^: p-value from Chi-square test.
